# Investigating the Intervention Parameters of Endogenous Paired Associative Stimulation (ePAS)

**DOI:** 10.3390/brainsci11020224

**Published:** 2021-02-12

**Authors:** Gemma Alder, Nada Signal, Alain C. Vandal, Sharon Olsen, Mads Jochumsen, Imran Khan Niazi, Denise Taylor

**Affiliations:** 1 Health and Rehabilitation Research Institute, Auckland University of Technology, Auckland 0627, New Zealand; nada.signal@aut.ac.nz (N.S.); sharon.olsen@aut.ac.nz (S.O.); imran.niazi@nzchiro.co.nz (I.K.N.); denise.taylor@aut.ac.nz (D.T.); 2 Department of Statistics, University of Auckland, Auckland 1142, New Zealand; alain.vandal@auckland.ac.nz; 3 Ko Awatea, Counties Manukau Health, Auckland 2025, New Zealand; 4 Department of Health Science and Technology, Aalborg University, 9000 Aalborg, Denmark; mj@hst.aau.dk; 5 Centre for Chiropractic Research, New Zealand College of Chiropractic, Auckland 1060, New Zealand

**Keywords:** paired associative stimulation, movement-related cortical potential, neuromodulation, neural plasticity, rehabilitation (MeSH), stroke (MeSH), factorial design, linear mixed regression

## Abstract

Advances in our understanding of neural plasticity have prompted the emergence of neuromodulatory interventions, which modulate corticomotor excitability (CME) and hold potential for accelerating stroke recovery. Endogenous paired associative stimulation (ePAS) involves the repeated pairing of a single pulse of peripheral electrical stimulation (PES) with endogenous movement-related cortical potentials (MRCPs), which are derived from electroencephalography. However, little is known about the optimal parameters for its delivery. A factorial design with repeated measures delivered four different versions of ePAS, in which PES intensities and movement type were manipulated. Linear mixed models were employed to assess interaction effects between PES intensity (suprathreshold (Hi) and motor threshold (Lo)) and movement type (Voluntary and Imagined) on CME. ePAS interventions significantly increased CME compared to control interventions, except in the case of Lo-Voluntary ePAS. There was an overall main effect for the Hi-Voluntary ePAS intervention immediately post-intervention (*p* = 0.002), with a sub-additive interaction effect at 30 min’ post-intervention (*p* = 0.042). Hi-Imagined and Lo-Imagined ePAS significantly increased CME for 30 min post-intervention (*p* = 0.038 and *p* = 0.043 respectively). The effects of the two PES intensities were not significantly different. CME was significantly greater after performing imagined movements, compared to voluntary movements, with motor threshold PES (Lo) 15 min post-intervention (*p* = 0.012). This study supports previous research investigating Lo-Imagined ePAS and extends those findings by illustrating that ePAS interventions that deliver suprathreshold intensities during voluntary or imagined movements (Hi-Voluntary and Hi-Imagined) also increase CME. Importantly, our findings indicate that stimulation intensity and movement type interact in ePAS interventions. Factorial designs are an efficient way to explore the effects of manipulating the parameters of neuromodulatory interventions. Further research is required to ensure that these parameters are appropriately refined to maximise intervention efficacy for people with stroke and to support translation into clinical practice.

## 1. Introduction

Advances in our understanding of neural plasticity have prompted the emergence of non-invasive neuromodulatory interventions [1]. These interventions have the potential to facilitate adaptive neural plasticity in partially disrupted neural networks and promote motor recovery following neurological injury such as stroke [2]. Paired associative stimulation (PAS) is an example of a non-invasive neuromodulatory intervention that is based on the principles of Hebbian-associative plasticity [3,4]. PAS involves the repeated temporal pairing of two stimuli, usually a single pulse of electrical stimulation to a peripheral nerve with a single pulse of transcranial magnetic stimulation (TMS) over the contralateral primary motor cortex (M1) [5]. PAS induces a rapid change in corticomotor excitability (CME) of the corticomotor projections from the M1 to the target muscle. The inter-stimulus interval (ISI) of the paired stimuli influences whether CME is increased or decreased [5,6]. (For reviews see [7,8,9].) The literature has proposed that these effects resemble spike-timing-dependent neural plasticity [10,11,12,13,14].

In contrast to PAS, endogenous paired associative stimulation (ePAS) is an intervention that substitutes the exogenous TMS stimulation with an endogenous movement-related signal. This endogenous signal is derived from electroencephalography (EEG) and is known as the movement-related cortical potential (MRCP). The MRCP is a slow negative potential observed during the preparation and execution of both imagined and voluntary movements [15,16]. The MRCP commences approximately 1.5–2 s prior to and peaks around the onset of movement [15,16]. When the MRCP is produced in response to externally cued movements (i.e., where there are two contingent cues, a “Warning” and a “Go” cue), it is also referred to as the contingent negative variation (CNV) [17]. Multiple cortical and subcortical neural substrates give rise to the generation of the MRCP; these include the supplementary motor area, cingulate motor areas, premotor cortex, prefrontal cortex, primary motor cortex, primary somatosensory cortex, thalamus, basal ganglia, and cerebellum [15,16,18,19,20]. Whilst the MRCP neural substrates are similar for the generation of externally cued and self-paced movements, the literature suggests that the dorsal premotor cortex plays a critical role in externally cued movements compared to those that are self-paced [21].

Typically, ePAS involves the repeated pairing of a single pulse of electrical stimulation to a peripheral nerve with the MRCP, such that the peripheral afferent stimulus arrives during the peak negativity (PN) of the MRCP [22,23]. Like PAS, there is some suggestion that the neuromodulatory effects of ePAS are dependent on the ISI, because CME is increased when the peripheral afferent stimulus is hypothesised to arrive in the M1 during the PN of the MRCP [22]. A single session of ePAS has been shown to increase CME for up to 60 min post-intervention in healthy participants performing imagined movement [22,23,24,25,26,27] and up to 30 min post-intervention in people with subacute [28] and chronic stroke [29] attempting voluntary movement. Improvements in motor function [28,30] and locomotor abilities have also been noted following ePAS interventions in people with chronic stroke [29].

Previous research has shown that ePAS can be delivered with various combinations of afferent stimulation (peripheral nerve stimulation, muscle stimulation, robotic-assisted passive movement) and endogenous motor activation (imagined or voluntary movement). To date, there has been little systematic exploration of the most effective method of delivering ePAS [22,31,32,33]. Given its potential to promote neural plasticity following stroke, identifying the most effective way in which to deliver the ePAS intervention is an important step prior to its translation into clinical populations and implementation in clinical practice.

Two intervention parameters that may influence the effectiveness of ePAS are (1) the peripheral electrical stimulation (PES) intensity and (2) the movement type: voluntary or imagined. With regards to PES intensity, research to date has delivered ePAS with the electrical stimulation set to motor threshold [22,23,24,25,28,31] or at 110% of the motor threshold [27]. However, neuroimaging and neurophysiological research that has investigated PES alone suggests that stimulation delivered above motor threshold (suprathreshold) is more likely to result in increased CME, compared with stimulation delivered at or below the motor threshold [34,35,36,37]. Therefore, it seems reasonable to investigate whether ePAS is more effective when delivered using suprathreshold PES compared to motor threshold PES. With regards to movement type, ePAS research in healthy participants has largely utilised an MRCP derived from imagined movement as the endogenous signal [22,23,24,25,26,31]. Whilst there are similarities in cortical activation between voluntary and imagined movement [38], voluntary movement calls upon more complex cortical networks [39], whereas imagined movement engages inhibitory networks to prevent the occurrence of voluntary movement [40,41]. Imagined movements generate lower levels of cortical activation compared to voluntary movements, as evidenced by MRCPs with lower amplitudes [42,43], lower levels of M1 activation on neuroimaging, and reduced CME [44,45,46,47,48,49]. When considering translation into clinical populations, it is also worth noting that motor imagery skills are often limited in people with stroke [36]. Furthermore, unlike voluntary movements, imagined movements do not provide internal feedback on performance, which is crucial for motor learning [50]. In light of this literature, it is vital to investigate whether ePAS delivered during voluntary movement yields larger increases in CME than ePAS delivered during imagined movements.

When exploring the effect of manipulating both PES intensity and movement type, it is important to consider their interaction. The literature indicates that greater increases in CME are seen when PAS or PES is combined with voluntary movement rather than with no movement [36,51,52,53]. Additionally, PES or PAS combined with voluntary movement is more effective than voluntary movement alone [52,53,54]. This suggests that the two parameters (electrical stimulation and movement type) may interact with one another to produce an enhanced or additive effect on CME. This might indicate that during ePAS, manipulating either PES intensity or movement type could individually influence intervention efficacy, and that manipulating both parameters together may produce an even greater effect. If the combined effect of suprathreshold PES + voluntary movement is greater than the addition of the two individual effects alone, this would be considered ‘super-additive’. It is possible that the delivery of ePAS using suprathreshold stimulation and voluntary movement might yield a super-additive effect.

The primary aim of this study was to investigate whether ePAS delivered using single pulses of suprathreshold PES, paired with MRCPs produced during voluntary ankle dorsiflexion movements, would yield a super-additive increase in CME in healthy adults, and whether this effect would be maintained for 45 min post-intervention. The secondary aims of this study were to investigate whether (a) ePAS using suprathreshold PES paired with voluntary dorsiflexion movement would yield the greatest effect on CME compared to all other interventions, (b) ePAS interventions that delivered suprathreshold PES intensities would yield greater effects on CME than those that delivered motor threshold PES or did not deliver PES (control), and (c) ePAS interventions that involved voluntary dorsiflexion movements would yield greater effects on CME than those involving imagined movements.

## 2. Materials and Methods

### 2.1. Study Design

**Figure 1:**
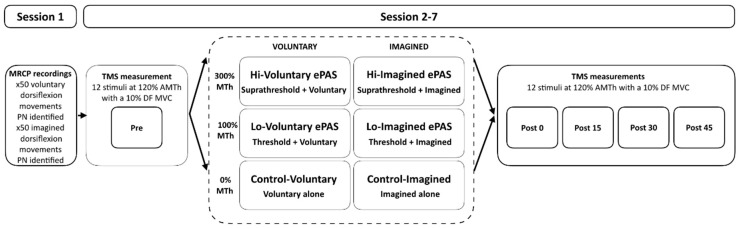
Schematic illustration of the factorial study design with six intervention conditions. MRCP, movement-related cortical potential; DF, dorsiflexor; PN, peak negativity; TMS; transcranial magnetic stimulation; AMTh, active motor threshold; MTh motor threshold; MVC, maximum voluntary contraction.

This study employed a factorial design comprising six interventions with repeated measures (refer to Figure 1). Factorial designs provide an efficient method to investigate the interaction effects of treatment parameters (in this case, stimulation intensity and movement type) and differentiate them from super-additive, additive, or sub-additive treatment effects [55]. A super-additive effect indicates that the combination is greater than the additive effects of the two treatment parameters alone. A sub-additive effect indicates that the combination is less than the additive effects of the two treatment parameters alone.

Participants completed a baseline testing session in which their MRCP was recorded during both voluntary and imagined ankle dorsiflexion movements. This was followed by six intervention sessions, which were completed in a randomised order and separated by at least 48 h. Randomisation was restricted so that no two sequences were identical. Interventions included (1) Hi-Voluntary ePAS: suprathreshold PES and voluntary dorsiflexion; (2) Hi-Imagined e-PAS: suprathreshold PES and imagined dorsiflexion; (3) Lo-Voluntary e-PAS: motor threshold PES and voluntary dorsiflexion; (4) Lo-Imagined e-PAS: motor threshold PES and imagined dorsiflexion; (5) Control-Voluntary: voluntary dorsiflexion alone; and (6) Control-Imagined: imagined dorsiflexion alone. TMS-derived measures of CME were recorded using single-pulse TMS immediately before and at 0, 15, 30, and 45 min following each intervention by a blinded assessor.

### 2.2. Sample Size Calculation

The study was powered to detect a super-additive effect size of 130 µV, corresponding to a Cohen’s effect size of 0.55 for the primary hypothesis. Simulations were predicated on the most relevant available literature [51], where TMS-derived measurements were recorded from a resting muscle and expressed as mean motor-evoked potential (MEP) amplitudes. Simulations assumed a within-participant correlation ranging between 0.35 and 0.85 and a standard deviation (SD) at a baseline of 0.23. To achieve a statistical power of 80%, 25 participants were required to participate in all six interventions.

### 2.3. Participants

Healthy adults aged over 18 years with no known neurological conditions volunteered for the study. Participants were excluded if they were taking medications that may have affected central nervous system excitability. Prior to the study, all participants completed a TMS screening eligibility questionnaire [56] and provided written consent. Participants were asked to abstain from strenuous exercise and caffeine consumption prior to data collection. The study was approved by the Auckland University of Technology Ethics Committee (15/270) and took place at the Health and Rehabilitation Research Institute at Auckland University of Technology, Auckland, New Zealand.

### 2.4. Experimental Set-Up and Procedures

#### 2.4.1. Session 1

##### MRCP Recordings

During session 1, MRCPs were derived from the EEG recorded during 50 repetitions of visually cued voluntary ankle dorsiflexion movement and 50 repetitions of visually cued imagined ankle dorsiflexion movement. The order of these two recordings was pseudo-randomised and separated by a 10-min rest period. Participants were seated with approximately 100° of hip flexion and the legs supported in 25° knee flexion with the ankles in a relaxed, slightly plantarflexed position. A 40-channel EEG cap (Quik-cap, Ag/AgCl electrodes Compumedics, Neuroscan, Dresden, Germany) was positioned with the CZ electrode positioned halfway between the nasion and the inion and halfway between each tragus. A sterile blunt needle was used to lightly abrade the scalp and administer conductive gel to the FP1, F3, FZ, F4, C3, CZ, C4, P3, PZ, and P4 electrodes (according to the international 10–20 system). A reference electrode was positioned over the mastoid process, and a ground electrode was placed on the forehead. Impedance of less than 5 kΩ was maintained.

Participants were familiarised with the visual cue displayed on a computer monitor that was customised in MATLAB software 7.13 (MathWorks, Inc., Natick, MA, USA, 2010. The visual cue prompted participants to (1) focus on the screen, (2) prepare for the ankle movement by watching a moving cursor, (3) execute either a ballistic voluntary or imagined dorsiflexion movement with their right ankle at a specified time-point and hold for 1 s, and (4) rest. The length of focus and rest periods varied (2–3 s and 6–8 s, respectively). Participants completed two sets of 25 repetitions for both voluntary and imagined movement conditions, while continuous EEG was recorded via a 40-channel EEG amplifier (NuAmps, Compumedics Neuroscan, Dresden, Germany) and sampled at 500 Hz with 32-bit accuracy (SCAN software, Compumedics Neuroscan, Dresden, Germany).

##### MRCP Feature Extraction

Continuous EEG signals were imported into MATLAB software 8.5 (MathWorks, Inc., Natick, MA, USA), where individual EEG channels were band-pass filtered from 0.05 to 5 Hz with a second-order zero-phase Butterworth filter. All channels excluding FP1 were spatially filtered using a large Laplacian filter to acquire a single virtual channel with CZ as the centre electrode [57]. The virtual channel was separated into 50 4.5 s epochs (3 s before the cue to move and 1.5 s after) [22,25,31,32]. Each of the 50 epochs was visually inspected and manually rejected if there was no evidence of a progressive negative shift preceding the cue or if FP1 electrooculographic activity surpassed 70 mV [22,25]. The remaining epochs were averaged, and the most negative point of the signal was selected as the PN.

#### 2.4.2. Sessions 2–7

Each session was conducted at a similar time of day, and sessions were separated by a minimum of 48 h. Participants adopted the same seated position as described in session 1. The six different interventions were dose-matched (number of repetitions n = 50) and lasted approximately 15 min. Participants followed the same visual cue described in session 1.

#### Outcome Measure—Corticomotor Excitability (CME)

The primary outcome measure was TMS-induced motor evoked potentials (MEPs) of the tibialis anterior (TA) muscle; this was assessed with single-pulse TMS during a 10% maximal voluntary contraction (MVC) of the dorsiflexor muscles to provide an indication of CME [58]. MEPs were recorded pre-intervention and at 0, 15, 30, and 45 min post-intervention. TMS procedures were replicated at each of the six sessions as per the International Federation of Clinical Neurophysiology (IFCN) guidelines [58].

For recording MEPs, electromyography (EMG) surface electrodes (Blue Sensor N, Ambu, Ballerup, Denmark) were placed over the muscle belly of the right TA muscle and a reference electrode was positioned over the right tibia based on SENIAM guidelines [59]. Prior to EMG electrode placement, the skin was shaved, abraded, and cleaned with alcohol to reduce impedance. If impedance exceeded 5 kΩ, skin preparation and electrode placement were repeated.

Prior to CME measurements, each participant’s dorsiflexor MVC was established. Fastening belts were secured around the right foot and ankle and both hips to maintain a stable position. Participants were instructed to dorsiflex their right ankle as hard as possible for 3–5 s and were provided with loud vocal encouragement and continuous visual feedback via an oscilloscope (TDS014B, Tektronix, Auckland, New Zealand). Participants performed three MVC contractions, each followed by a 2-min rest. Isometric dorsiflexion force data were collected using a single axis load cell (Model PTASP6-E, Precision Transducers Limited, Auckland, New Zealand, capacity 300 kg and error <0.02%), sampled at 100 Hz via a data acquisition board (Micro 1401, CED, Cambridge, UK) and processed using Signal software (CED, Cambridge, UK). The MVC was established by measuring the peak amplitude of the largest of the three trials and deducting the mean baseline value recorded at rest [60]. A 10% MVC value was then calculated. This level of contraction was used during TMS measurements based on its sensitivity to changes in motor-evoked potential (MEP) amplitude [61].

For the application of TMS, participants wore a fitted cap with 1 × 1 cm gridlines relative to the vertex. Single-pulse TMS was administered with a Magstim 200 using a monophasic pulse and posterior-anterior current flow [58] via a double cone coil (10 cm outer diameter per wing, Magstim Company Limited, Dyfed, UK), as per recommendations for lower limb M1 stimulation [62]. Initially, the junction of the coil was positioned approximately 0.5–1 cm lateral to CZ [63,64]. Stimulation intensity was initially delivered at 30% of the stimulator output and increased in increments of 5%. The coil position was systematically adjusted to determine the ‘hotspot’, which was defined as the location where the largest TA MEP was induced at the lowest stimulation intensity. Grid references for the hotspot were recorded to maintain accurate positioning of the coil in subsequent applications. Participants were then required to generate a 10% dorsiflexor MVC that matched a visual target on an oscilloscope, during which the active motor threshold (AMT) was established; the AMT was defined as the lowest stimulus intensity required to produce a minimum of 5 TA MEPs out of 10 stimuli with peak–peak amplitudes of ≥100 µV [58]. At each measurement time-point, 12 single-pulse TMS stimuli were delivered at 120% of AMT during a 10% MVC. Stimuli were delivered every 6–8 s, and participants were encouraged to focus their attention on the oscilloscope target. TA EMG data were amplified to 1000 Hz (AMT-8, Bortec Biomedical, Canada) and sampled at 2000 Hz via a data acquisition board (Micro 1401, CED, Cambridge, UK) and Signal software (CED, Cambridge, UK). The methodological quality of the TMS measurement procedures for this study were evaluated in accordance with the TMS Quality Checklist [65], which is reported in Supplementary Material (Table S1).

#### ePAS Interventions

The peripheral electrical stimulation (DS7A, Digitimer Limited, Hertfordshire, UK) was applied via two additional surface electrodes (Blue Sensor N, Ambu, Ballerup, Denmark) that were positioned on the skin over the right deep common peroneal nerve (dCPN), approximately 2–4 cm anteriorly and inferiorly to the head of the fibula, with the cathode placed proximally. The optimal stimulation location was determined by palpating for TA tendon movement without the presence of palpable synergistic and antagonistic activity [25,31,32]. Once the optimal location was identified, the motor threshold (MTh) was determined: this was defined as the lowest stimulation intensity (mA) required to elicit a palpable flicker in the TA tendon [25,32].

The level of suprathreshold PES intensity was established from pilot work. It was deemed important to select a tolerable PES intensity to minimise antidromic effects that could negatively influence the intervention [66]. A visual analogue scale (VAS) was utilised to rate the discomfort of various PES intensities, and piloting indicated that an intensity of 300% MTh did not exceed a rating of 5/10 (*n* = 5, mean intensity 33 mA, mean VAS 4/10). Of the four ePAS interventions, the two interventions Hi-Voluntary and Hi-Imagined delivered a PES intensity at 300% of MTh to elicit a substantial TA muscle contraction. The other two interventions, Lo-Voluntary and Lo-Imagined, delivered a PES intensity at 100% of MTh to elicit a palpable flicker in the TA tendon (see Figure 1).

During the delivery of ePAS, participants completed 50 repetitions of either voluntary or imagined movement, while 50 single pulses (1 ms) of PES were delivered to the dCPN. Each 1 ms PES pulse was delivered 50 ms prior to the PN of the participant’s average MRCP for the corresponding movement condition. The 50 ms represents the average conduction time from the dCPN to the M1 [22].

#### Control Interventions

**Figure 2:**
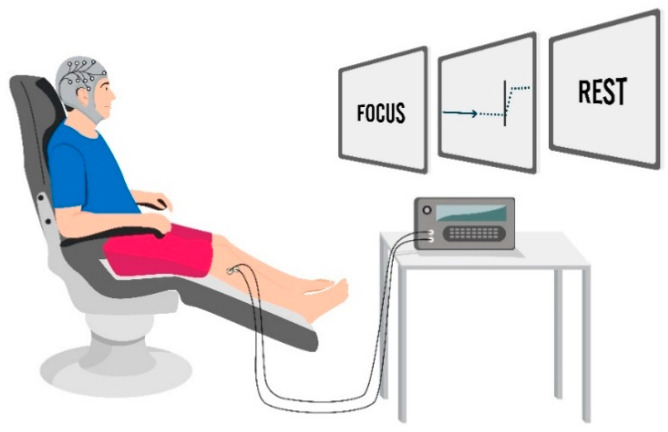
Intervention set-up. Participants observed the visual cue to prompt the timing of either voluntary or imagined dorsiflexion whilst receiving either suprathreshold (300%) stimulation, motor threshold stimulation (100%), or no stimulation (0%) to the right deep common peroneal nerve.

For the two control interventions (Control-Voluntary and Control-Imagined), participants performed either 50 visually cued voluntary dorsiflexion movements or 50 visually cued imagined dorsiflexion movements of the right ankle. Sham PES was delivered concurrently (set to 0% MTh). An illustration of the laboratory set-up is displayed in Figure 2.

### 2.5. Data Processing and Analysis

TA EMG data were processed using Signal software (CED, Cambridge, UK). A predefined processing criterion was established to address contaminated EMG responses at each of the measurement time-points and is provided in Supplementary Material (Table S2). Less than 5% of the EMG data were discarded. CME outcomes were processed using a customised script developed in MATLAB 2015a to identify four MEP parameters: (1) absolute MEP amplitude (µV); (2) absolute MEP area (µV/ms); (3) relative MEP amplitude (% change); and (4) relative MEP area (% change). The decision to investigate MEP area was based on literature that suggests it may be more sensitive to changes due to the polyphasic nature of MEPs in the lower limb [67,68]. The decision to investigate both absolute and relative MEP data was consistent with a number of previous neuromodulation studies [9,27]. Absolute MEP amplitude values were established by extracting the peak-to-peak amplitude of each individual MEP observation. Absolute MEP area values were established by measuring the area of rectified EMG activity of each individual MEP for 30 ms, starting 2 ms before its onset. MEP onset was defined as the first point where EMG activity exceeded 2 standard deviations of mean background EMG activity for more than 2 ms on an averaged waveform for each measurement time-point. The background area was established by rectifying individual EMG signals and calculating the area of a 70 ms window that terminated 2 ms prior to the stimulation artefact. Individual MEP amplitudes and areas were used for the absolute MEP statistical analysis. For the relative MEP analysis, the individual MEP amplitudes and areas were averaged to give a mean MEP for each participant at each time-point. Relative MEP amplitude and MEP area % change values were calculated as follows: [(post-pre)/pre] × 100.

### 2.6. Statistical Analysis

A detailed account of the pre-specified statistical analysis plan is provided in the Supplementary Material. The inferential framework selected for the primary and secondary analysis was linear mixed regression modelling, which provides greater statistical efficiency and minimises the risk of type-I error compared to a repeated measures ANOVA [69]. The large size of the datasets (>7800 individual MEP observations in the absolute MEP data and >580 observations in the relative MEP data) renders concerns about non-normality of the residuals secondary, in spite of the dependence between the observations, if we extend the arguments of Lumley and colleagues regarding linear regression to linear mixed regression [70]. Analyses were carried out using the package lme4 [71] in R (R Core Team) and SAS/STAT™ software. A blind review was carried out to identify covariates for adjustment and the covariance structure of potential models. The baseline covariates MVC and AMTh were tested for adjustment in the models (using blinded treatment codes to adjust for treatment) and using a 5% significance threshold to decide on inclusion. Absolute and relative pre-intervention MEP amplitude and MEP area values were also tested in the same manner. The blind model selection was based on Akaike’s information criterion [72]. An assessment of residual covariance structure and heteroscedasticity across the blind intervention groups in the retained models was also carried out. Missing data were assumed to be missing at random; under this assumption, the linear mixed regression analysis adequately allayed selection bias from missing data [73]. The actual models retained for the analysis were the versions of the blind models with an interaction term between movement type and stimulation intensity to address the primary hypothesis. For the analysis of absolute datasets, the model was identical for both MEP amplitude and MEP area data. A decision was made *a priori* to refrain from applying corrections of adjustment for multiplicity due to the explanatory nature of this study [74]. Type III errors for terms and interactions were obtained against a null hypothesis that set to zero all higher-order interactions involving the target term or interaction. Mean MEP effect sizes (µV and µV/ms) and 95% confidence intervals were also calculated. Means and standard deviations are reported as the mean ± SD for participant characteristics, MRCP data, and PES intensities. Processing and analysis of relative MEP data are reported in Supplementary Material.

## 3. Results

### 3.1. Participant Characteristics

The mean age of the 25 participants was 28 ± 7 years (range 19–52), and 15 participants were female. Three potential participants were excluded from the study, one due to a recent skull fracture and two due to routinely taking medication that may have altered excitability of the central nervous system.

#### 3.1.1. MRCP

**Figure 3:**
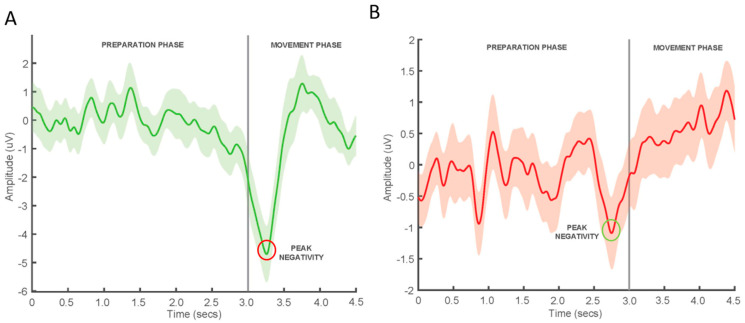
Average MRCPs with 95% confidence intervals obtained from one participant (**A**) performing voluntary ankle dorsiflexion and (**B**) performing imagined ankle dorsiflexion. The vertical line at the 3-s mark corresponds to the visual cue to move.

During the processing of the 50 MRCP epochs, on average, 5 ± 2 and 9 ± 6 epochs, for voluntary and imagined movement conditions, respectively, were manually rejected due to eye blinks or artefacts. The timing of the PN of the averaged MRCP occurred at a mean of 13 ± 67 ms after the cue in the voluntary movement condition and 4 ± 155 ms after the cue in the imagined condition. Figure 3 provides examples of averaged MRCPs with 95% confidence intervals obtained from an individual performing voluntary ankle dorsiflexion (A) and imagined movement ankle dorsiflexion (B).

#### 3.1.2. PES Intensities (mA)

The dCPN PES was delivered at the following intensities during each of the four ePAS interventions: Hi-Voluntary 37 ± 18 mA, Hi-Imagined 33 ± 16 mA, Lo-Voluntary 14 ± 8 mA, and Lo-Imagined 11 ± 4 mA.

#### 3.1.3. Baseline Corticomotor Excitability (CME)

Baseline raw MEP values for absolute MEP amplitude and MEP area are reported in Table 1.

### 3.2. Study Findings

Due to the explanatory nature of this study, a large number of results were generated from the models. Within this paper, we present the key findings, and all other results are provided in the Supplementary Material. The results for baseline covariates, model interactions, and main effects for absolute MEP amplitude and absolute MEP area are provided in Table 2.

#### 3.2.1. Primary Findings

**Figure 4:**
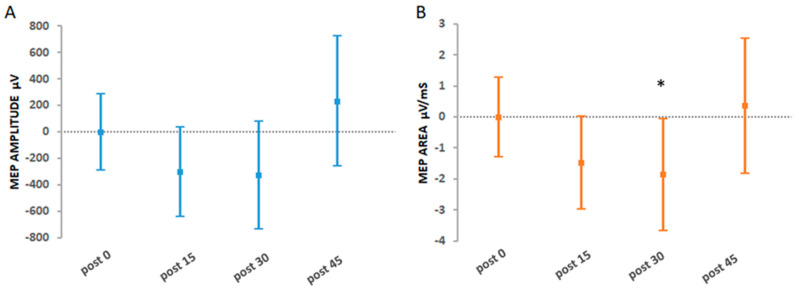
(**A**) MEP amplitude and (**B**) MEP area adjusted estimates for the primary analysis investigating super- or sub-additive effects for the Hi-Voluntary intervention at each post-intervention time-point. Error bars depict 95% CIs; * indicates significant effects (*p* < 0.05).

The primary aim was to investigate whether Hi-Voluntary would yield a super-additive increase in CME, which was maintained for 45 min post-intervention. Table 2 presents the interactions for the effects of Hi-Voluntary whereby the statistical model with the interaction term (super- or sub-additivity) was compared to the statistical model with no interaction (additivity) for the levels of stimulation intensity and movement type. Figure 4 displays the results of the primary analysis for absolute MEP amplitude (µV) and absolute MEP area (µV/ms) at each post time-point.

For the Hi-Voluntary intervention, the analysis revealed an interaction between suprathreshold stimulation intensity versus no stimulation and voluntary versus imagined movement for both absolute MEP amplitude (*p* = 0.0001) and MEP area (*p* < 0.00005). The analysis did not reveal a super-additive effect. However, a significant sub-additive effect was observed for absolute MEP area 30 min post-intervention (−1.87 µV/ms 95% CI [−3.68, −0.06], *p* = 0.042). There was no significant interaction for suprathreshold stimulation versus threshold stimulation and voluntary versus imagined movement for absolute MEP amplitude (*p* = 0.139) and MEP area (*p* = 0.108).

An unplanned post hoc analysis investigated whether Lo-Voluntary also had a sub-additive effect on CME. This analysis confirmed a significant interaction between threshold stimulation intensity (100% MTh) versus no stimulation (0% MTh) and voluntary versus imagined movement for absolute MEP amplitude (*p* = 0.0013) and MEP area (*p* = 0.0004). A significant sub-additive effect occurred at 15 min post-intervention for the absolute MEP amplitude (−505.6 µV 95% CI [−871.4, −139.8], *p* = 0.006) and MEP area (−3.21.6 µV/ms 95% CI [−4.9, −1.48], *p* = 0.0003). This sub-additive effect extended to 30 min post-intervention for absolute MEP area (−2.79 µV/ms 95% CI [−5.01, −0.57], *p* = 0.014).

#### 3.2.2. Secondary Findings: The Effects of Hi-Voluntary Compared to All Other Interventions

**Figure 5:**
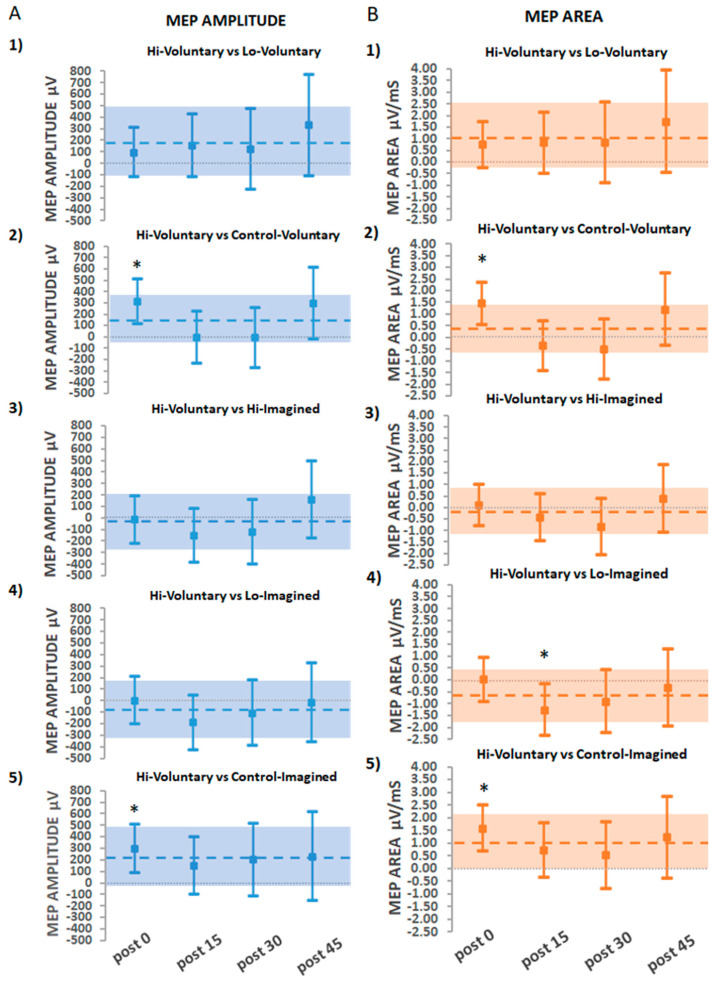
Estimated adjusted effect differences in absolute units between Hi-Voluntary and each intervention on (**A**) MEP amplitude and (**B**) MEP area, at each post-intervention time point and averaged over time. Error bars depict 95% CIs. Dashed lines depict estimates averaged over time with a shaded bar for 95% CIs; * indicates significant effects (*p* < 0.05).

A secondary aim of the study investigated whether ePAS using suprathreshold PES paired with voluntary dorsiflexion movement (Hi-Voluntary) would yield the greatest increase in CME compared to all other interventions. These comparisons are illustrated in Figure 5A for absolute MEP amplitude (µV) and Figure 5B for absolute MEP area (µV/ms).

Absolute MEP amplitudes were significantly greater for Hi-Voluntary compared to Control-Voluntary immediately post-intervention (311 µV 95% CI [109, 511.8], *p* = 0.002) and when compared to Control-Imagined immediately post-intervention (295.5 µV 95% CI [87.37, 503.6], *p* = 0.005). Comparisons for Hi-Voluntary with each of the other ePAS interventions (Lo-Voluntary, Hi-Imagined, and Lo-Imagined) were non-significant at all time-points (*p* > 0.1 in all comparisons).

Results for absolute MEP area replicated those of absolute MEP amplitude, except in the case of Hi-Voluntary compared to Lo-Imagined, where a greater significant effect was observed at 15 min post-intervention for the Lo-Imagined intervention (−1.28 µV/ms 95% CI [−2,37, −0.19], *p* = 0.021).

#### 3.2.3. Secondary Findings: Stimulation Intensity

**Figure 6:**
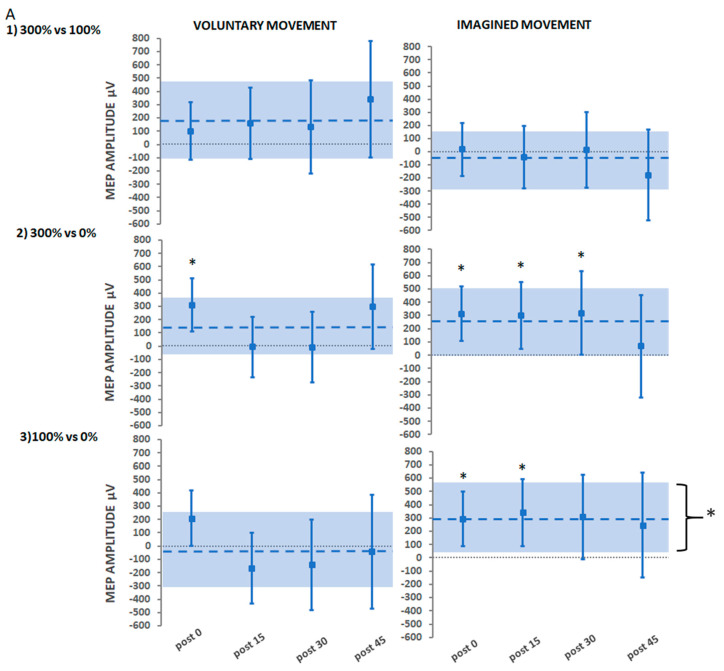

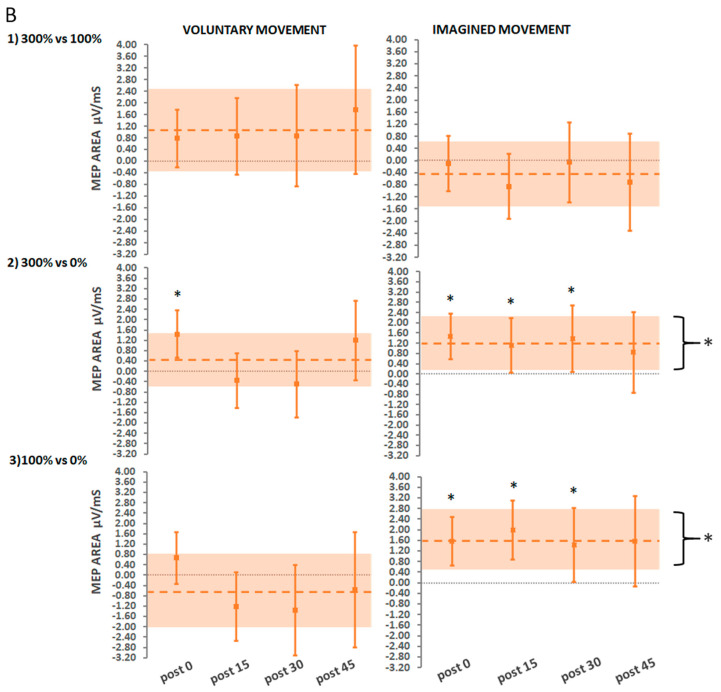
Estimated adjusted effect differences between stimulation intensity levels delivered during voluntary movement and imagined movement on (**A**) MEP amplitude and (**B**) MEP area, at each post-intervention time-point and averaged over time. Error bars depict 95% CIs. Dashed lines depict estimates averaged over time with a shaded bar for 95% CIs. * indicates significant effects (*p* < 0.05).

A further secondary aim of the study was to investigate whether treatment interventions that delivered suprathreshold PES intensities (Hi-Voluntary, Hi-Imagined) would yield greater increases in CME than those that delivered threshold intensities (Lo-Voluntary, Lo-Imagined) and no stimulation (Control-Voluntary, Control-Imagined). These comparisons are illustrated in Figure 6A for absolute MEP amplitude (µV) and Figure 6B for absolute MEP area (µV/ms).

#### Suprathreshold Stimulation (Hi) vs. Threshold Stimulation (Lo)

Comparisons for ePAS interventions delivered with suprathreshold compared to threshold stimulation for either voluntary or imagined movement conditions (Hi-Voluntary vs. Lo-Voluntary, Hi-Imagined vs. Lo-Imagined) were not significant for absolute MEP amplitude or MEP area (*p* > 0.1 in all comparisons).

#### Suprathreshold Stimulation vs. No Stimulation

Comparisons determined that absolute MEP amplitudes were significantly greater for Hi-Voluntary compared to Control-Voluntary immediately post-intervention only (311 µV 95% CI [109, 511.8], *p* = 0.002). For the imagined movement interventions, absolute MEP amplitudes were significantly greater for Hi-Imagined compared to Control-Imagined immediately (312 µV 95% CI [105.2, 518.8], *p* = 0.003), 15 min (299 µV 95% CI [47.35, 551.2], *p* = 0.020) and 30 min post-intervention (319 µV 95% CI [5.58,632.4], *p* = 0.046). Results for absolute MEP area replicated those of absolute MEP amplitude, except in the case of the averaged over time comparison that determined a significantly greater increase for Hi-Imagined compared to Control-Imagined (1.2 µV/ms 95% CI [0.14, 2.58], *p* = 0.026).

#### Threshold Stimulation vs. No Stimulation

There were no significant differences in absolute MEP amplitude for any of the comparisons made for Lo-Voluntary and Control-Voluntary (*p* > 0.05 in all comparisons). However, for Lo-Imagined, there were significant increases in absolute MEP amplitude when compared to Control-Imagined immediately post-intervention (295 µV 95% CI [89.6, 500.7], *p* = 0.004), 15 min post-intervention (339.9 µV 95% CI [86.28, 593.6], *p* = 0.009), and averaged over time (297 µV 95% CI [36.3, 558], *p* = 0.026). The results for absolute MEP area replicated those of absolute MEP amplitude, except in the 30 min post-intervention time-point comparison that revealed a significantly greater increase for Lo-Imagined compared to Control-Imagined (1.4 µV/ms 95% CI [0.03, 2.81], *p* = 0.043).

#### 3.2.4. Secondary Findings: Movement Type

**Figure 7:**
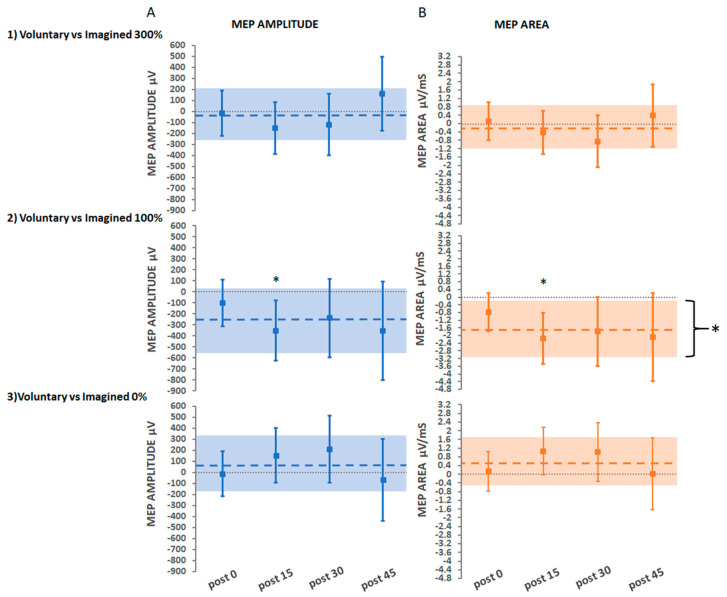
Estimated adjusted effect differences between voluntary and imagined movement at each stimulation intensity level on (**A**) MEP amplitude and (**B**) MEP area, at each post-intervention time-point and averaged over time. Error bars depict 95% CIs. Dashed lines depict averaged over time estimates with a shaded bar for 95% CIs; * indicates significant effects (*p* < 0.05).

The secondary aim was to investigate whether treatment interventions that involved voluntary dorsiflexion movement (Hi-Voluntary, Lo-Voluntary, Control-Voluntary) would yield greater increases in CME than treatment interventions that involved imagined movements (Hi-Imagined, Lo-Imagined, Control-Imagined). Comparisons made between voluntary and imagined movement at different intensities of stimulation are illustrated in Figure 7A for absolute MEP amplitude (µV) and Figure 7B for absolute MEP area (µV/ms).

#### Voluntary vs. Imagined Movement at Suprathreshold Stimulation

Comparisons for the Hi-Voluntary and Hi-Imagined interventions were non-significant for absolute MEP amplitude and MEP area (*p* > 0.1 in all comparisons).

#### Voluntary vs. Imagined Movement at Threshold Stimulation

Comparisons determined that absolute MEP amplitude was significantly greater for Lo-Imagined compared with Lo-Voluntary at 15 min post-intervention only (−352 µV 95% CI [−626.2, −78.29], *p* = 0.012). The results for absolute MEP area replicated those of absolute MEP amplitude, except in the case of the averaged over time comparison that determined a significantly greater increase for Lo-Imagined compared to Lo-Voluntary (−1.7 µV/ms 95% CI [−3.18, −0.22], *p* = 0.025).

#### Voluntary vs. Imagined Movement with No Stimulation

Comparisons for the Control-Voluntary and Control-Imagined interventions were non-significant for absolute MEP amplitude and MEP area (*p* > 0.05 in all comparisons).

## 4. Discussion

This ePAS study is the first to systematically unpack the effect of manipulating the parameters of stimulation intensity and movement type in an effort to enhance intervention efficacy prior to translation to clinical populations and implementation in rehabilitation practice. To support the discussion of the results, we first provide an overview of the main effects of the four ePAS interventions investigated and then focus on the primary hypothesis exploring the interaction effects of Hi-Voluntary ePAS. Following this, the interpretation of the interaction effects is supported by systematically exploring the effects of the two intervention parameters within the factorial design: PES intensity and movement type.

### 4.1. ePAS Intervention Efficacy

The ePAS interventions Hi-Voluntary, Hi-Imagined, and Lo-Imagined were all significantly more effective at increasing CME than their respective control interventions. These findings are in keeping with previous research investigations of Lo-Imagined ePAS interventions in healthy people [22,23,24,25,26,27]. This study extends those findings by demonstrating that Hi-Imagined and Hi-Voluntary ePAS interventions are also effective at increasing CME in healthy people. However, Lo-Voluntary ePAS was not more effective at increasing CME than Control-Voluntary. This finding is in contrast with a previous study that assessed the effect of a Lo-Voluntary intervention delivered just above the motor threshold (110% MTh) [27]. In that study, the intervention effects were not significantly different from the Control-Voluntary intervention immediately post-intervention, but they were significantly increased at 30 min post-intervention [27]. It is notable in our findings that the duration of the effect differed across ePAS interventions. In both the Hi-Imagined and Lo-Imagined interventions, an increase in CME was seen for up to 30 min post-intervention, whereas in the Hi-Voluntary intervention, the effect did not last beyond the immediate post-intervention time point. Previous research in Lo-Imagined ePAS interventions reported durations of effects lasting between 30 and 60 min post-intervention for both absolute and relative MEP amplitude data when recorded from a resting muscle [24,25,26,33]. Explanation for the differences between the four ePAS interventions in both the magnitude and duration of effects are explored below through the interpretation of these findings in relation to both movement type and stimulation intensity.

### 4.2. Hi-Voluntary ePAS

The primary objective of this study was to investigate whether an ePAS intervention that paired suprathreshold PES with the MRCP during voluntary movement (Hi-Voluntary) would yield a super-additive increase in CME of the TA muscle in healthy adults for up to 45 min post-intervention. Our findings did not confirm this hypothesis. The MEP area analysis did reveal a significant interaction between stimulation intensity and movement type, illustrating a *sub-additive* effect at 30 min post-intervention. This finding suggests that the combination of suprathreshold stimulation and voluntary movement (Hi-Voluntary) produced a smaller increase in CME than the sum of these two parameters. Given that CME was significantly greater following Hi-Voluntary than following Control-Voluntary, it can be asserted that while the Hi-Voluntary intervention loses some of the effect of stimulation intensity and voluntary movement when paired, the intervention is more effective at increasing CME than performing voluntary movement alone (Control-Voluntary). A potential explanation for why we did not find a super-additive effect for the Hi-Voluntary intervention could be that the intervention parameters require further refinement and optimisation. Further work is required to fully elucidate the mechanisms of action of this intervention.

### 4.3. Stimulation Intensity

For the secondary analyses of the factor ‘stimulation intensity’, it was hypothesised that interventions that delivered suprathreshold stimulation (Hi-Voluntary, Hi-Imagined) would yield greater increases in CME than those that delivered threshold PES (Lo-Voluntary, Lo-Imagined) or no stimulation (Control-Voluntary, Control-Imagined). This hypothesis was not supported, as there was no difference between suprathreshold and threshold stimulation. However, there were differences between stimulation (Hi-Voluntary, Hi-Imagined, Lo-Imagined) and no stimulation (Control-Voluntary, Control-Imagined).

The post hoc analysis investigating the interaction effect of the Lo-Voluntary intervention revealed that, akin to Hi-Voluntary, Lo-Voluntary had a *sub-additive* effect at some time-points post-intervention. It is notable that the magnitude of the sub-additive effect was greater for the Lo-Voluntary intervention than for the Hi-Voluntary intervention. In voluntary movement conditions, the low-intensity afferent volley generated in the Lo-Voluntary intervention may be subsumed by the endogenous motor cortex activation generated during the preparation and execution of voluntary movement. Jochumsen and colleagues [32] showed that the pairing of motor threshold nerve stimulation with the MRCPs from voluntary movement (Lo-Voluntary) was no more effective at increasing CME in healthy people than either voluntary movement or PES alone. Their findings showed that to be effective, the voluntary movement needed to be paired with muscle stimulation delivered at motor threshold [32]; muscle stimulation may have produced a larger afferent volley, less likely to be subsumed by endogenous M1 activity. These findings might reflect the differences in the stimulation frequency, current density, and motor unit recruitment observed when stimulating muscle versus a nerve [32,75,76,77] and warrant further investigation into the PES intensity applied during ePAS interventions.

In contrast to our findings in healthy people, Lo-Voluntary ePAS applied to people with stroke has been shown to be more effective than attempted voluntary movement alone for increasing CME [28,29] and muscle strength [30]. Many people with stroke have lower levels of motor cortex activation [64,78,79], which might be less likely to subsume the afferent stimulation. However, studies investigating the effects of PES alone in people with stroke have reported that higher intensities of stimulation are more effective at increasing CME, reducing impairment, and improving function [80,81,82]. The ePAS intervention has not yet been applied at stimulation intensities greater than the motor threshold in people with stroke. It is possible that, if well tolerated, higher PES intensities may be required to maximise the effects of ePAS in people with stroke.

### 4.4. Movement Type

For the secondary analyses of the factor of ‘movement type’, it was hypothesised that interventions that involved voluntary movement (Hi-Voluntary, Lo-Voluntary, Control-Voluntary) would yield greater increases in CME than imagined movements (Hi-Imagined, Lo-Imagined, Control-Imagined). Our findings did not support this hypothesis, demonstrating no difference in CME between voluntary and imagined movement conditions except for the Lo-Imagined intervention. When compared to the Lo-Voluntary intervention, Lo-Imagined yielded larger effects at 15 min post-intervention (MEP amplitude and MEP area) and when all time-points were averaged (MEP area). The effect of the Lo-Imagined intervention has been demonstrated in previous ePAS studies in healthy people [22,23,24,25,26,27]. This may be linked to the concept of subsumed afferent input discussed above.

In this study, we used the same ISI across all participants for each of the four ePAS interventions [22]. It is possible that the optimal temporal pairing between PES and cortical activity, and thus the ISI, differs between imagined and voluntary movement. Imagined movement involves motor preparation and activation of M1, but the final command to activate the descending corticospinal neurons is inhibited [40]. Thus, the timing at which the afferent volley arrives at M1 may interact differently with facilitatory and inhibitory networks in voluntary versus imagined movement. The ISI is known to be critical in determining whether PAS paradigms result in an increase or decrease in CME [5,6,83]. A recent systematic review that examined the efficacy of PAS on lower limb CME and the influence of stimulation parameters found that increases in CME were largest when selecting an ISI of 40–55 ms or an optimised ISI based on the individualised somatosensory evoked potential latency [9]. However, little prior work has been done to inform the optimisation of the ISI during ePAS [22], and no studies have investigated the optimal ISI during voluntary movement ePAS conditions. Future ePAS research should therefore investigate the optimal ISI for both the individual and the movement type.

A final consideration for the delivery of ePAS using either voluntary or imagined movement is its potential application to stroke rehabilitation. Whilst the imagined movement condition was more effective when the PES was delivered at the motor threshold (Lo-Imagined), we have previously acknowledged the potential benefits of giving higher intensity stimulation to people with stroke, and in this study, the combination of higher intensity stimulation with voluntary movement (Hi-Voluntary) produced significant effects. In addition, people with stroke might have difficulty performing motor imagery due to cortical damage [84], and voluntary movement has the additional benefit of providing internal feedback on performance, which is essential for motor learning [50]. Thus, from a clinical perspective, there are a number of potential benefits of delivering ePAS during voluntary movement, but further research is required to compare the effects of various combinations of stimulation intensity and movement type in people with stroke.

### 4.5. Strength and Limitations

To our knowledge, this is the first study to begin to unpack the effects of different intervention parameters of a neuromodulatory intervention using a factorial design. The findings of this study are strengthened by the use of a sound *a priori* statistical analysis plan, including the consideration of baseline covariates that might introduce variability in the data, and an analysis of both MEP amplitude and area and measures of change (absolute and relative). A number of considerations are required when interpreting the results.

First, the study was powered to detect changes in MEP amplitude, not MEP area, yet some findings were only observed in MEP area data. Our choice of MEP amplitude as a primary outcome measure was driven by the availability of data for the sample size calculation [22]. However, MEP area was considered an important outcome, as evidence suggests it may be more sensitive to changes due to the polyphasic nature of MEPs in the lower limb [67,85].

Second, whilst the sample size was powered to detect a super-additive effect corresponding to a Cohen’s effect size of 0.55, our data featured a much larger between-participant standard deviation at the pre-intervention time-point than the work used to power this study [51], resulting in an effect size of just 0.15. It is possible that this large variability was related to the use of active MEP measurements [86] in our study, in contrast to the resting MEPs used in the study that informed the sample size calculation [22]. MEPs are difficult and sometimes impossible to elicit in the resting muscle of people with stroke [64,78,79], and therefore, we chose to record active MEPs in our study. This would enable replication of this study in the stroke population and minimise selection bias in future work. In our study, the magnitude of change in active MEPs may have been smaller than the measurement error, hindering the ability to see interaction effects. We have provided between-participant standard deviations for active MEP amplitude and active MEP area measures, which can be used to inform future calculations of standardised effect sizes.

Third, whilst we made every effort to ensure high methodological quality during TMS measurement procedures (see Supplementary Material: TMS Quality Checklist) the use of a standard hand-held TMS coil could reduce the ability to reliably locate the M1 representation of interest [87]. The use of neuronavigation systems with neuroimaging to track the coil and head position during TMS measurement procedures has demonstrated improved spatial accuracy of cortical localisation [88,89], decreased variability in trial-to-trial MEPs [90], and more timely TMS procedures [91] compared to the standard hand-held coil method. Therefore, if available, the use of neuronavigation should be considered to strengthen the methodological quality of TMS measurement procedures.

Fourth, the method used in this study to identify the timing of the peak negativity involved an offline manual method, where the MRCP data were averaged from pre-recorded voluntary and imagined movements. This method has been used extensively in the literature to determine the timing of the temporal pairing in efficacious ePAS interventions [22,24,25,26,27,28,29,30,33,92]. However, if the temporal pairing is a key factor that dictates the success of the ePAS intervention, the use of online detection for each MRCP trial could improve the accuracy of individual pairings in real time and maximise the effects of the intervention [23,26,92].

Finally, a factorial design enables the researcher to evaluate multiple intervention parameters and identify potential interactions between these parameters whilst maintaining statistical efficiency [55]. However, for pragmatic reasons, we did not include control conditions of suprathreshold stimulation only (Hi) and threshold stimulation only (Lo) in the factorial design. Whilst a number of previous ePAS [22,23,33], PAS [51,93], and PES [52] studies have failed to show any effects of stimulation alone, this decision limited our ability to fully unpack the effect of the different intervention parameters.

### 4.6. Future Recommendations

In order to advance our understanding of the optimal delivery of ePAS, we recommend that future research consider the following:

- Factorial designs should be used to explore the interaction effects of different intervention parameters in neuromodulatory interventions; parameters could include stimulation intensity, movement type, ISI, and the number of stimuli.
- To support translation into clinical practice, this work and similar factorial designs should be undertaken in people with stroke. These studies should not only explore the neurophysiological effects of the intervention but also assess changes in impairment and functional outcomes.
- Using TMS measurements from both resting and active muscle would allow comparison to previous work and enable the researcher to consider the impact of measurement error on the findings.
- The baseline covariates AMTh and MVC were considered and suitably adjusted for in the factorial models for the primary analysis of this study. Future research should also consider interactions with baseline covariates as part of a pre-planned blind model selection process. This may shed light on how baseline covariates modulate responses to neuromodulatory interventions.

## 5. Conclusions

Factorial designs are an efficient way to explore the effects of manipulating the parameters of neuromodulatory interventions. The present study systematically unpacked the effect of manipulating PES intensity and movement type in ePAS interventions. Our findings make several contributions to the current evidence. First, the findings are consistent with previous research investigations that support the excitatory effect of Lo-Imagined ePAS interventions on CME in healthy people. Second, this study extends those findings by demonstrating that delivering suprathreshold PES stimulation intensities during both Hi-Imagined and Hi-Voluntary ePAS interventions is also effective. Third, our findings indicate an interaction effect between intervention parameters; this effect was sub-additive for Hi-Voluntary and Hi-Imagined ePAS interventions. From a clinical perspective, we have discussed the potential benefits of delivering Hi-Voluntary ePAS. However, to support translation into clinical practice, further research is necessary to evaluate the efficacy of different configurations of stimulation intensity and movement type in people with stroke. Further factorial study designs should be considered to determine the most effective ePAS intervention parameters for people with stroke.

## Figures and Tables

**Table 1:** Baseline raw motor-evoked potential (MEP) values for absolute MEP amplitude and MEP area.

	MEP Amplitude (µV)	MEP Area (µV/ms)
**Mean**	1730	7.2
**Within SD**	1060	4.6
**Between SD**	839	3.8

**Table 2:** Observed significance of baseline covariates and main and interaction effect estimates of absolute MEP amplitude and MEP area.

		MEP Amplitude (µV)	MEP Area (µV/ms)
	Numerator *df*	*p* Value	*p* Value
**Baseline covariates**			
AMTh	1	0.0001 *	0.0001 *
MVC	1	0.06	0.04 *
**Main effects and interactions**			
Stimulation intensity	16	<0.00005 *	<0.00005 *
Movement type	12	0.002 *	0.001 *
Time	18	0.009 *	0.0004 *
Stimulation intensity × Movement type	8	0.0005 *	0.0001 *
Stimulation intensity × Time	12	0.002 *	<0.00005 *
Movement type × Time	9	0.001 *	0.002 *
Stimulation intensity × Movement type × Time	6	0.0004 *	0.0003 *
**Hi-Voluntary intervention**			
Super-/sub-additivity of suprathreshold stimulation overall, voluntary vs. imagined	8	0.0005 *	0.0001 *
Super-/sub-additivity of suprathreshold stimulation vs. no stimulation and voluntary vs. imagined	4	0.0001 *	<0.00005 *
Super-/sub-additivity of suprathreshold stimulation vs. threshold and voluntary vs. imagined	4	0.139	0.108
**Lo-Voluntary intervention**			
Super-/sub-additivity of threshold stimulation vs. no stimulation and voluntary vs. imagined	4	0.0013 *	0.0004 *

*df*, degrees of freedom; AMTh, active motor threshold; MVC, maximum voluntary contraction; MTh, motor threshold. Significant effects (*p* < 0.05) with *.

## Supplementary Materials

The following are available online at https://www.mdpi.com/2076-3425/11/2/224/s1, Table S1: TMS Quality Checklist scores; Table S2: Predefined TA EMG data processing criteria; Table S3: Estimated adjusted Super/sub-additive effects in absolute units of stimulation intensity levels (vs no stimulation) and Voluntary movement (vs Imagined movement) on MEP amplitude and MEP area, at each post-baseline time point; Table S4:Estimated adjusted effect differences in absolute units between Hi-Voluntary and each intervention on MEP amplitude and MEP area, at each post-baseline time point and averaged over time; Table S5a: Estimated adjusted effect differences in absolute units between stimulation intensity levels delivered during voluntary movement and imagined movement on MEP amplitude, at each post-baseline time-point and averaged over time; Table S5b: Estimated adjusted effect differences in absolute units between stimulation intensity levels delivered during voluntary movement and imagined movement on MEP area, at each post-baseline time-point and averaged over time; Table S6: Estimated adjusted effect differences in absolute units between voluntary and imagined movement at each stimulation intensity level on MEP amplitude and MEP area, at each post-baseline time-point and averaged over time; Table S7: Observed significance of estimated baseline covariate, main and interaction effects for Relative MEP amplitude and MEP area; Table S8: Estimated adjusted extra-additive effects in percentage points from baseline of stimulation intensity levels (vs no stimulation) and Voluntary movement (vs Imagined movement) on MEP amplitude and MEP area; Table S9: Estimated adjusted effect differences in percentage points from baseline between Hi-Voluntary and each intervention on the MEP amplitude and MEP area; Table S10a: Estimated adjusted effect differences in percentage points from baseline between stimulation intensity levels delivered during voluntary movement and imagined movement on MEP amplitude; Table S10b: Estimated adjusted effect differences in percentage points from baseline between stimulation intensity levels delivered during voluntary movement and imagined movement on MEP area; Table S11: Estimated adjusted effect differences in percentage points from baseline between movement types at each stimulation intensity level on MEP amplitude and MEP area.
