# Supplementary material for: Investigating the Intervention Parameters of Endogenous Paired Associative Stimulation (ePAS)
Source: Brain Sci. 2021 Feb 12;11(2):224. doi: 10.3390/brainsci11020224 (PMC7918620; doi:10.3390/brainsci11020224)
Supplement: Supplementary file 1 [file brainsci-11-00224-s001.pdf]

brain sciences

MDPI

Article

Investigating the Intervention Parameters of Endogenous Paired Associative Stimulation (ePAS)

Gemma Alder [1,*], Nada Signal [1], Alain C. Vandal [2,3], Sharon Olsen [1], Mads Jochumsen [4], Imran Khan Niazi [1,4,5] and Denise Taylor [1]

[1] Health and Rehabilitation Research Institute, Auckland University of Technology, Auckland 0627, New Zealand; nada.signal@aut.ac.nz (N.S.); sharon.olsen@aut.ac.nz (S.O.); imran.niazi@nzchiro.co.nz (I.K.N.); denise.taylor@aut.ac.nz (D.T.)

[2] Department of Statistics, University of Auckland, Auckland 1142, New Zealand; alain.vandal@auckland.ac.nz

[3] Ko Awatea, Counties Manukau Health, Auckland 2025, New Zealand

[4] Department of Health Science and Technology, Aalborg University, 9000 Aalborg, Denmark; mj@hst.aau.dk

[5] Centre for Chiropractic Research, New Zealand College of Chiropractic, Auckland 1060, New Zealand

* Correspondence: gemma.alder@aut.ac.nz

Supplementary Material

Table S1. TMS Quality Checklist scores.

	Reported	Controlled
**Participant factors**		
Age of participants	1	1
Gender of participants	1	NA
Handedness / Footedness of participants	1	1
Participants prescribed medication	1	1
Use of CNS active drugs	1	1
Presence of neurological/psychiatric disorders in healthy participants	1	1
Any medical conditions	1	1
History of specific repetitive motor activity	0	0
**Methodological factors**		
Position and contact of EMG electrodes	1	1
Amount of relaxation/contraction of target muscles	1	1
Prior motor activity of the muscles to be tested	1	1
Level of relaxation of muscles other than those being tested	NA	0
Coil type (size and geometry)	1	1
Coil orientation	1	1
Direction of induced current in the brain	1	1
Coil location and stability (with or without neuro-navigation system)	1	1
Type of stimulator used (e.g., brand)	1	1
Stimulation intensity	1	1

Pulse shape (monophasic or biphasic)	1	1
Determination of optimal hotspot	1	1
The time between MEP trials	1	1
Time between days of testing	1	1
Subject attention (level of arousal) during testing	1	1
Method of determining threshold (active/resting)	1	1
Number of MEP measures made	1	1
**Paired pulse only**		
Intensity of test pulse	NA	NA
Intensity of conditioning pulse	NA	NA
Inter-stimulus interval	NA	NA
**Analytical factors**		
Method of determining MEP size during analysis	1	1
Size of unconditioned MEP	NA	NA
**Totals**	24	23
% Score	96%	92%
**Overall % Score**	**94%**	

1 = yes, 0 = no, NA = non-applicable.

Table S2. Predefined TA EMG data processing criteria.

1	The first EMG response was removed from each time point due to participants often being startled from the sensation and/or anticipation of the first TMS pulse.
2	EMG responses were removed if there was no stimulation artefact visually present corresponding to the TMS trigger.
3	EMG responses will be removed if silent EMG or excessively large/erratic voluntary muscle activity was present within the 70ms preceding the stimulation artefact or if during data collection the participant was not at their 10% force line when the TMS pulse was triggered.

Statistical analysis plan

1. Analysis sets and datasets

1.1. Missingness at random assumption

It is assumed that missing data are missing at random (MAR). The planned linear mixed effects analysis will adequately account for missing data under this assumption [Carpenter 2007].

1.2. Datasets

There will be two datasets used in the analysis. One will be the absolute dataset, consisting of the absolute values of the outcomes pre-intervention, immediately post-intervention, and at 15, 30, and 45 minutes post-intervention (normally, 5 time-points). All measurements at all time-points will be retained. The second one will be called the relative dataset and will consist of the relative change from the pre-intervention measure immediately post-intervention and at 15, 30, and 45 minutes post-intervention. These relative changes will be computed from the mean of the measurements at each time-point and a weight corresponding to the number of observations retained post-intervention to

compute the mean at each post-intervention time point. The relative change was calculated as follows (post-pre)/pre × 100.

2. Study outcomes and baseline covariates

2.1. Cortico-motor excitability (CME) outcomes

CME outcomes are measured pre-intervention and at 0, 15, 30, and 45 minutes post-intervention. They consist of:

(1) Absolute MEP amplitude values
(2) Absolute MEP area values
(3) MEP amplitude % change values
(4) MEP area % change values

2.2. Baseline covariates

The covariates collected pre-intervention consist of maximum voluntary contraction (MVC) and active motor threshold percentage (AMT). These covariates have been tested for adjustment in the models during a blind review (using the blinded treatment codes to adjust for treatment), using a 5% significance threshold to decide on inclusion. Pre-intervention MEP amplitude and MEP area values have also been tested in the same manner.

3. Structure of analyses

3.1. Notes on the data

We distinguish between Blind Intervention and the Blind Intervention Group in the absolute data only.

Blind Intervention takes on the values "None", "A", "B", "C", "D", "E", and "F", with "None" applying in all pre-intervention observations. It is used to define fixed effects.

The Blind Intervention Group takes on values "A", "B", "C", "D", "E", and "F" and refers to the intervention applied in a whole session, including the pre-intervention measurements. It is used to define random effects.

Only the Blind Intervention Group is used in the relative data, as the pre-intervention observations are not included in the analyses.

3.2 Inferential framework

The inferential framework selected is linear mixed modelling. The large size of the datasets (over 7300 observations in the absolute data and over 580 observations in the relative data) render concerns about non-normality of the secondary residuals, in spite of the dependence between the observations, if we extend the arguments of Lumley and colleagues [Lumley, Diehr, Emerson, and Chen 2002] regarding linear regression to linear mixed regression. Analyses were carried out using the package *lme4* [Bates, Machler, Bolker and Walker 2014] in *R (R* Core Team 2018) *and SAS/STAT™ software.*

3.3 Blinded selection of model

During the blind review, the model to be used will be selected from amongst 144 models (absolute data) or 288 models (relative data) defined with the core elements listed below. A final assessment of residual covariance structure and heteroscedasticity across the Blind Intervention Groups in the retained model will be carried out.

<u>Core covariate:</u>

- Blind Intervention: None, A, B, C, D, E, F (None is applied to the pre-intervention data)

<u>Core random effects:</u>

- Participant random intercept: P01,…,P025
- Participant and Blind Intervention Group interaction random intercept: P01*A, P01*B, etc.

The model also involves the following alternative components.

Alternative covariates:

(1) Time-point: either time as a factor (PRE, POST0, POST15, POST30, POST45), time as continuous (PRE=0,…,POST45=4), or time as continuous as well as its square
(2) Time-point and Blind Intervention interaction: either none, or in interaction with time as a factor, or in interaction with time as a continuous covariate
(Note: For covariates only, we do not mix categorical and continuous in the same model.)
(3) MVC: Present or not
(4) AMT: Present or not
(5) Baseline MEP value: Present or not (relative data only)
(6) Order of the intervention in the cross-over: Present or not

Alternative random effects

(7) Participant random slope for time as continuous: Present or not
(8) Participant and Blind Intervention Group interaction random slope for time as continuous: Present (only if 7. is present) or not

Notes:

Time was taken as continuous in random effect regardless of fixed effects. Model selection was automated up to the selection of items 1–8 with the use of the package *lme4 [58]* in *R* (R Core Team, 2018). Further covariance investigation was carried out using PROC MIXED, part of the SAS/STAT software. In case item 7 was retained, alternative formulations for the variance structure were investigated (*R*-side covariance structure), with time as a continuous index and instances of participant-interventions as the subject. These covariance structures were:

- compound symmetry
- heterogeneous compound symmetry
- autoregressive of order 1
- autoregressive moving average of order (1, 1)
- spatial exponential
- spatial Gaussian
- spatial power covariance.

In all cases, heterogeneity of the variance parameters by treatment group was investigated, always using AIC as a criterion. Under failure to converge, the model was deemed inadequate without further investigation.

3.4. Retained models

3.4.1. Absolute data

The final model was selected based on Akaike's information criterion and corresponded to the following model for both MEP amplitude and MEP area.

Retained fixed effects:

- Blind Intervention
- Time as a factor
- Blind Intervention in interaction with time
- AMT
- MVC

Retained random effects:

- Participant random intercept
- Participant in interaction with Blind Intervention Group random intercept and random time slope

Other variance parameters:

- Variances were found to be heterogeneous across Blind Treatment Groups.

3.4.2. Relative data

The final model for the relative data was selected based on Akaike's information criterion and corresponded to the following model for both relative MEP amplitude and MEP area.

Retained fixed effects:
- Blind Intervention
- Absolute baseline value

Retained random effects:
- Participant random intercept
- Autoregressive moving average of order 1,1, with continuous time as the index and participant-time as the subject

Other variance parameters:
- Variance parameters were found to be heterogeneous across Blind Treatment Groups.

3.5. Primary analysis

3.5.1. Translation to unblinded model

The actual model retained for the primary analysis is the factorial version of the retained model. Movement type takes on the values "None", "Real", or "Imagined"; intensity takes on the values "NA", "0%", "100%", or "300%". The values "None" for movement type and "NA" for intensity only apply in the pre-intervention phase for Tx equal to "None".

3.5.2. Absolute data

Retained fixed effects:
- Movement type
- Intensity
- Time as a factor
- Movement type in interaction with time
- Intensity in interaction with time
- Three-way interaction of Movement type, Intensity and time
- AMT
- MVC

Retained random effects:
- Participant random intercept

Other variance parameters:
- Participant in interaction with Blind Intervention Group random intercept and random time slope.
- Heterogeneous variances across interventions.

3.5.3. Relative data

Retained fixed effects:
- Movement type
- Intensity
- Movement type in interaction with intensity
- Baseline absolute value

Retained random effects:
- Participant random intercept

Other variance parameters:

- Autoregressive moving average of order 1, in the measurement time ordering, with participant-intervention as the subject; heterogeneous variance parameters across interventions.

Table S3. Estimated adjusted super-/sub-additive effects in absolute units of stimulation intensity levels (vs. no stimulation) and Voluntary movement (vs. Imagined movement) on MEP amplitude and MEP area at each post-baseline time-point.

	Adjusted estimate MEP Amplitude µV Δ (95% CIs)	p-value	Adjusted estimate MEP Area µV/ms Δ (95% CIs)	p-value
Primary analysis: super-/sub-additivity, intensity suprathreshold vs. no stimulation and real vs. imaginary movement				
POST0	-1.2 (-289.2,86.8)	0.99	-0.02 (-1.31,1.26)	0.97
POST15	-305.3 (-643,32.34)	0.076	-1.49 (-2.99,0.011)	0.051
POST30	-327.9 (-735.7,79.83)	0.11	-1.87 (-3.68,-0.06)	**0.042***
POST45	229.7 (-262.9,722.3)	0.35	0.355 (-1.83,2.53)	0.74
super-/sub-additivity, intensity threshold vs. no stimulation and real vs. imaginary movement				
POST0	-85.64 (-378.9,207.6)	0.56	-0.91 (-2.25,0.438)	0.18
POST15	-505.6 (-871.4,-139.8)	**0.006***	-3.21 (-4.9,-1.48)	**0.0003***
POST30	-448.1 (-909.8,13.68)	0.05	-2.79 (-5.01,-0.57)	**0.014***
POST45	-287.3 (-860.3,285.7)	0.32	-2.13 (-4.91,0.645)	0.13

*Δ: Difference; Significant effects (p <0.05) are in bold text with **

Table S4. Estimated adjusted effect differences in absolute units between Hi-Voluntary and each intervention on MEP amplitude and MEP area at each post-baseline time-point and averaged over time.

	Adjusted estimate MEP Amplitude µV Δ (95% CIs)	p-value	Adjusted estimate MEP Area µV/ms Δ (95% CIs)	p-value
**Hi-Voluntary vs. Lo-Voluntary**				
POST0	101 (-114.1,316.8)	0.35	0.78 (-0.22,1.778)	0.12
POST15	160 (-111.8,431)	0.24	0.863 (-0.45,2.177)	0.19
POST30	132 (-218.3,481.8)	0.45	0.869 (-0.87,2.607)	0.32
POST45	339 (-100.6,778.2)	0.12	1.767 (-0.44-3,975)	0.11
Averaged over time	183 (-104.8,470.5)	0.20	1.07 (-0.36,2.503)	0.13
**Hi-Voluntary vs. Control-Voluntary**				
POST0	311 (109,511.8)	**0.002***	1.443 (0.527,2.359)	**0.002***
POST15	-6 (-232.9,220.9)	0.95	-0.36 (-1.42,0.703)	0.50
POST30	-9 (-275.2,257.3)	0.94	-0.5 (-1.78,0.783)	0.44
POST45	298 (-18.7,614.2)	0.06	1.195 (-0.35,2.744)	0.12
Averaged over time	148 (-67.37,364.1)	0.17	0.445 (-0.6,1.489)	0.39
**Hi-Voluntary vs. Hi-Imagined**				
POST0	-16.58 (-222.7,189.5)	0.87	0.112 (-0.8,1.026)	0.81
POST15	-151.9 (-386.5,82.69	0.20	-0.43 (-1.47,0.609	0.41
POST30	-119.9 (-399.6,159.9)	0.39	-0.85 (-2.09,0.384)	0.17
POST45	160.1 (-174.8,495)	0.34	0.384 (-1.1,1.865)	0.60
Averaged over time	-32.07 (-260.3,196.1)	0.78	0.2 (-1.21,0.812)	0.69
**Hi-Voluntary vs. Lo-Imagined**				
POST0	0.341 (-204.4,205.1)	0.99	0.011 (-0.91,0.93)	0.98
POST15	-192.6 (-429,43.82)	0.10	-1.28 (-2.37,-0.19)	**0.021***
POST30	-108.3 (-394.5,178)	0.45	-0.91 (-2.25,0.433)	0.18
POST45	-18.15 (-363,326.7)	0.91	-0.34 (-1.97,1.294)	0.68
Averaged over time	-79.67 (-313.1,153.8)	0.49	-0.63 (-1.72,0.466)	0.25
**Hi-Voluntary vs. Control-Imagined**				

POST0	295.5 (87.37,503.6)	**0.005***	1.579 (0.667,2.492)	**0.000***
POST15	147.3 (-103.5,398.2)	0.24	0.7 (-0.37,1.775)	0.20
POST30	199.1 (-114,512.2)	0.20	0.517 (-0.8,1.836)	0.43
POST45	228 (-156.8,612.8)	0.23	1.224 (-0.38,2.829)	0.13
Averaged over time	217.5 (-38.85,473.8)	0.094	1.005 (-0.07,2.082)	0.066

*Δ: Difference; Significant effects (p <0.05) are in bold text with **

Table S5a. Estimated adjusted effect differences in absolute units between stimulation intensity levels delivered during voluntary movement and imagined movement on MEP amplitude at each post-baseline time-point and averaged over time.

	Adjusted estimate MEP Amplitude μV Δ (95% CIs)	p-value	Adjusted estimate MEP Amplitude μV Δ (95% CIs)	p-value
**Voluntary**			**Imagined**	
**300% vs. 100%**				
POST0	101 (-114.1,316.8)	0.35	16.92 (-186.7-220.5)	0.87
POST15	160 (-111.8,431)	0.24	-40.68 (-278.4-197.1)	0.73
POST30	132 (-218.3,481.8)	0.45	11.6 (-275.2-298.4)	0.93
POST45	339 (-100.6,778.2)	0.12	-178.2 (-524-167.5)	0.30
Averaged over time	183 (-104.8,470.5)	0.20	-47.6 (-281.1-185.9)	0.68
**300% vs. 0%**				
POST0	311 (109,511.8)	**0.002***	312 (105.2-518.8)	**0.003***
POST15	-6 (-232.9,220.9)	0.95	299.3 (47.35-551.2)	**0.020***
POST30	-9 (-275.2,257.3)	0.94	319 (5.587-632.4)	**0.046***
POST45	298 (-18.7,614.2)	0.06	67.97 (-317.5-453.4)	0.72
Averaged over time	148 (-67.37,364.1)	0.17	249.6 (-6.64-505.8)	0.056
**100% vs. 0%**				
POST0	210 (-0.14,419.1)	0.050	295.1 (89.58,500.7)	**0.004***
POST15	-166 (-431.9,100.5)	0.21	339.9 (86.28,593.6)	**0.009***
POST30	-141 (-480.9,199.6)	0.41	307.4 (-11.79,626.5)	0.058
POST45	-41 (-468.1,385.9)	0.84	246.2 (-147.8,640.2)	0.21
Averaged over time	-35 (-312.9,243.9)	0.80	297.2 (36.28,558)	**0.026***

*Δ: Difference; Significant effects (p <0.05) are in bold text with **

Table S5b. Estimated adjusted effect differences in absolute units between stimulation intensity levels delivered during voluntary movement and imagined movement on MEP area at each post-baseline time-point and averaged over time.

	Adjusted estimate MEP Area μV/ms Δ (95% CIs)	p-value	Adjusted estimate MEP Area μV/ms Δ (95% CIs)	p-value
**Voluntary**			**Imagined**	
**300% vs. 100%**				
POST0	0.78 (-0.22,1.778)	0.12	-0.1 (-1.01,0.812)	0.82
POST15	0.863 (-0.45,2.177)	0.19	-0.85 (-1.93,0.228)	0.12
POST30	0.869 (-0.87,2.607)	0.32	-0.05 (-1.37,1.265)	0.936
POST45	1.767 (-0.44,3.975)	0.11	-0.72 (-2.32,0.881)	0.37
Averaged over time	1.07 (-0.36,2.503)	0.13	-0.43 (-1.51,0.643)	0.42
**300% vs. 0%**				
POST0	1.443 (0.527,2.359)	**0.002***	1.468 (0.566,2.369)	**0.001***
POST15	-0.36 (-1.42,0.703)	0.50	1.13 (0.063,2.197)	**0.038***
POST30	-0.5 (-1.78,0.783)	0.44	1.371 (0.074,2.668)	**0.038***

POST45	1.195 (-0.35,2.744)	0.12	0.84 (-0.73-2,414)	0.28
Averaged over time	0.445 (-0.6,1.489)	0.39	1.202 (0.146,2.258)	**0.026***
**100% vs. 0%**				
POST0	0.663 (-0.33,1.654)	0.18	1.568 (0.65,2.479)	**0.000***
POST15	-1.22 (-2.55,0.104)	0.070	1.982 (0.86,3.096)	**0.000***
POST30	-1.37 (-3.12,0.385)	0.12	1.424 (0.03,2.817)	**0.043***
POST45	-0.57 (-2.8,1.659)	0.60	1.56 (-0.15,3.274)	0.073
Averaged over time	-0.63 (-2.07,0.818)	0.38	1.634 (0.49,2.771)	**0.005***

*Δ: Difference; Significant effects (p <0.05) are in bold text with **

Table S6. Estimated adjusted effect differences in absolute units between voluntary and imagined movement at each stimulation intensity level on MEP amplitude and MEP area at each post-baseline time-point and averaged over time.

	**Adjusted estimate MEP Amplitude μV Δ (95% CIs)**	**p-value**	**Adjusted estimate MEP Area μV/ms Δ (95% CIs)**	**p-value**
**Voluntary vs. Imagined 300%**				
POST0	-16.58 (-222.7,189.5)	0.87	0.112 (-0.81.026)	0.81
POST15	-151.9 (-386.5,82.69	0.20	-0.43 (-1.47,0.609	0.41
POST30	-119.9 (-399.6,159.9)	0.39	-0.85 (-2.09,0.384)	0.17
POST45	160.1 (-174.8,495)	0.34	0.384 (-1.1,1.865)	0.60
Averaged over time	-32.07 (-260.3,196.1)	0.78	-0.2 (-1.21,0.812)	0.69
**Voluntary vs. Imagined 100%**				
POST0	-101 (-313.9,111.9)	0.35	-0.77 (-1.76,0.227)	0.12
POST15	-352.2 (-626.2,-78.29)	**0.012***	-2.15 (-3.49,-0.8)	**0.002***
POST30	-240 (-595.3,115.4)	0.18	-1.78 (-3.57,0.017)	0.052
POST45	-356.9 (-804.2,90.31)	0.11	-2.1 (-4.39,0.181)	0.070
Averaged over time	-262.5 (-554.1,28.99)	0.076	-1.7 (-3.18,-0.22)	**0.025***
**Voluntary vs. Imagined 0%**				
POST0	-15.38 (-217.7,186.9)	0.88	0.137 (-0.77,1.041)	0.76
POST15	153.4 (-91.9,398.7)	0.21	1.06 (-0.03,2.152)	0.056
POST30	208.1 (-93.97,510.1)	0.17	1.016 (-0.32,2.356)	0.13
POST45	-69.65 (-440,300.7)	0.70	0.029 (-1.61,1.667)	0.97
Averaged over time	69.11 (-176.8,315)	0.57	0.561 (-0.53,1.652)	0.30

*Δ: Difference; Significant effects (p <0.05) are in bold text with **

Table S7. Observed significance of estimated baseline covariate and main and interaction effects for relative MEP amplitude and MEP area.

	**Numerator *df***	**Relative MEP Amplitude (%) *P* value**	**Relative MEP Area (%) *P* value**
Baseline absolute value	1	**0.004***	**0.002***
Movement type	3	0.71	0.46
Stimulation intensity	4	0.17	0.12
Simulation intensity × Movement type	2	0.50	0.28

Significant effects (p <0.05) are in bold text with *

Table S8. Estimated adjusted extra-additive effects in percentage points from baseline of stimulation intensity levels (vs. no stimulation) and Voluntary movement (vs. Imagined movement) on MEP amplitude and MEP area.

Adjusted estimate MEP Amplitude – pct pt Δ (95% CIs)	p-value	Adjusted estimate MEP Area – pct pt Δ (95% CIs)	p-value
Super-/sub-additivity, intensity suprathreshold vs. no stimulation and real vs. imaginary movement			
-1.30 (-24.7,22.15)	0.91	-3.7 (-27.5,20.1)	0.76
Super-/sub-additivity, intensity threshold vs. no stimulation and real vs. imaginary movement			
-11.4 (-31.1,8.3)	0.25	-16.4 (-37.1,4.3)	0.12

*pct pt Δ: percentage point difference; Significant effects (p <0.05) are in bold text with **

Table S9. Estimated adjusted effect differences in percentage points from baseline between Hi-Voluntary and each intervention on the MEP amplitude and MEP area.

Contrast	Adjusted estimate MEP Amplitude – pct pt Δ (95% CIs)	p-value	Adjusted estimate MEP Area – pct pt Δ (95% CIs)	p-value
Hi-Voluntary vs. Lo-Voluntary	13.3 (-7.2,33.8)	0.20	13.3 (-6.1,32.7)	0.17
Hi-Voluntary vs. Control-Voluntary	13.4 (-5.7,32.4)	0.16	12.2 (-7.0,31.4)	0.20
Hi-Voluntary vs. Hi-Imagined	2.8 (-17.6,23.2)	0.78	1.4 (-18.4,21.3)	0.89
Hi-Voluntary vs. Lo-Imagined	6.0 (-15.0,27.1)	0.56	2.0 (-19.1,23.2)	0.85
Hi-Voluntary vs. Control-Imagined	17.5 (-2.6,37.6)	0.086	17.3 (-2.6,37.2)	0.087

*pct pt Δ: percentage point difference; Significant effects (p <0.05) are in bold text with **

Table S10a. Estimated adjusted effect differences in percentage points from baseline between stimulation intensity levels delivered during voluntary movement and imagined movement on MEP amplitude.

	Adjusted estimate MEP Amplitude – pct pt Δ (95% CIs)	p-value	Adjusted estimate MEP Amplitude – pct pt Δ (95% CIs)	p-value
	**Voluntary**		**Imagined**	
**300% vs. 100%**	13.3 (-7.2,33.8)	0.20	3.2 (-12.3,18.7)	0.68
**300% vs. 0%**	13.4 (-5.7,32.4)	0.16	14.7 (0.4,29.0)	**0.044***
**100% vs. 0%**	0.1 (-12.6,12.8)	0.99	11.5 (-3.8,26.8)	0.14

*pct pt Δ: percentage point difference; Significant effects (p <0.05) are in bold text with **

Table S10b. Estimated adjusted effect differences in percentage points from baseline between stimulation intensity levels delivered during voluntary movement and imagined movement on MEP area.

	Adjusted estimate MEP Area – pct pt Δ (95% CIs)	p-value	Adjusted estimate MEP Area - pct pt Δ (95% CIs)	p-value
	**Voluntary**		**Imagined**	
**300% vs. 100%**	13.3 (-6.1,32.7)	0.17	0.6 (-15.4,16.6)	0.94
**300% vs. 0%**	12.2 (-7.0,31.4)	0.20	15.9 (1.3,30.5)	**0.034***
**100% vs. 0%**	-1.1 (-14.0,11.7)	0.86	15.3 (-1.2,31.7)	0.068

*pct pt Δ: percentage point difference; Significant effects (p <0.05) are in bold text with **

Table S11. Estimated adjusted effect differences in percentage points from baseline between movement types at each stimulation intensity level on MEP amplitude and MEP area.

	**Adjusted estimated effect on MEP Amplitude – pct pt Δ (95% CIs)**	**p-value**	**Adjusted estimated effect on MEP Area - pct pt Δ (95% CIs)**	**p-value**
**Voluntary vs. Imagined 300%**	2.8 (-17.6,23.2)	0.78	1.4 (-18.4,21.3)	0.89
**Voluntary vs. Imagined 100%**	-7.3 (-23.0,8.4)	0.35	-11.3 (-27.1,4.5)	0.15
**Voluntary vs. Imagined 0%**	4.1 (-8.1,16.4)	0.50	5.1 (-8.7,18.9)	0.46

pct pt Δ: percentage point difference
